# Protective Effects of N-Acetylcysteine in Concanavalin A-Induced Hepatitis in Mice

**DOI:** 10.1155/2015/189785

**Published:** 2015-03-02

**Authors:** Chengfen Wang, Yujing Xia, Yuanyuan Zheng, Weiqi Dai, Fan Wang, Kan Chen, Jingjing Li, Sainan Li, Rong Zhu, Jing Yang, Qin Yin, Huawei Zhang, Junshan Wang, Jie Lu, Yingqun Zhou, Chuanyong Guo

**Affiliations:** Department of Gastroenterology, Shanghai Tenth People's Hospital, Tongji University of Medicine, Shanghai 200072, China

## Abstract

This study was designed to study the protective effects and mechanisms of N-acetylcysteine (NAC) in concanavalin A-induced hepatitis in mice. In this study, pretreatment with NAC ameliorated the histopathological changes and suppressed inflammatory cytokines in ConA-induced hepatitis. The expression of IL-2, IL-6, TNF-*α*, and IFN-*γ* was significantly reduced in the NAC-treated groups. NAC activated PI3K/Akt pathway and inhibited the activation of NF-*κ*B. Additionally, NAC reduced autophagosome formation, as assessed by detecting the expression of LC3 and Beclin 1. Our results demonstrate that NAC can alleviate ConA-induced hepatitis by regulating the PI3K/Akt pathway and reducing the late stages of autophagy. Our results described a new pharmaceutical to provide more effective therapies for immune hepatitis.

## 1. Introduction

Liver diseases, including viral hepatitis, toxic liver diseases, alcoholic liver diseases, and autoimmune hepatitis, represent a global health problem in humans. So far, only some kinds of the liver diseases can be managed pharmacologically [1, 2]. Therefore, new pharmaceuticals need to be developed and tested to provide more effective therapies in the clinic. However, the study of new pharmaceuticals requires proper animal models that are relevant to human liver diseases. The pathophysiology of liver disease is complex, and it depends upon humoral interorgan relationships, the integrity of metabolic pathways, and the highly sophisticated morphological organization of organs [3–5]. The activation of T cells has been established to be the initial trigger of most cases of autoimmune and viral hepatitis [6, 7]. These conditions are characterized by increased levels of aspartate transaminase (AST) and alanine transaminase (ALT) enzyme activities, and accompanied with lymphocyte activation and the inflammatory cytokines accumulation [8, 9]. In mice injected with the T-cell mitogenic plant lectin concanavalin A (ConA), T lymphocytes become activated. This process is consistent with a liver-specific inflammatory response that induces T-cell mediated hepatitis [9, 10]. Pathological studies have shown that the destruction of liver tissue mainly depends upon the infiltration and accumulation of many lymphocytes in hepatitis, principally CD4^+^T cells, natural killer T (NKT) cells, and Kupffer cells [10–12]. Additionally, proinflammatory cytokines also play an important role in hepatitis, such as tumor necrosis factor- (TNF-) *α*, interferon- (IFN-) *γ*, and interleukin- (IL-) 6 [13]. Nuclear factor-kappa B (NF-*κ*B) is a well-known transcription factor that can potently induce proinflammatory mediators' secretion during the development of acute liver disease. NF-*κ*B activity is mainly regulated by I*κ*B*α* and can be affected by the PI3K/Akt pathway. In summary, ConA-induced liver injury in mice is an appropriate model of immune hepatitis.

Autophagy, known as type II programmed cell death, is characterized as an intracellular protective mechanism by enveloping defective organelles by double-membrane vesicles formation termed autophagosomes which subsequently submitted to lysosomes to be degraded [14]. Paralleled with apoptosis, autophagy is another way of programmed cell death [15]. There are many regulators of autophagy, including mammalian target of rapamycin (mTOR), mTOR kinase, 5′-AMP-activated protein kinase (AMPK), eukaryotic initiation factor 2*α* (eIF2*α*), inositol-trisphosphate (IP3), and c-Jun-N-terminal kinase. Beclin 1, UVRAG, Vps34, Vps15, and Bif-1 also play important roles in autophagosome nucleation. One of the most important regulator pathway is the mTOR pathway, which could be modulated by PI3K/Akt pathway [16, 17]. Light chain 3 is known to be an important marker of autophagy expressed on autophagic vesicles. Beclin 1 is an essential component for the induction of autophagy. A certain extent of autophagy contributes to cellular homeostasis especially in stressed conditions such as nutrient restriction, hypoxia, and infection [14]. However, the role of autophagy in cells remains controversial. Highly accelerated autophagy has been reported in ischemia-induced neuronal cell death in the mouse brain. Moreover, autophagy plays a key role in the pathogenesis of myopathy [18].

N-Acetylcysteine (NAC) is a thiol and a precursor of both L-cysteine and glutathione (GSH) [19]. It is known to be a source of sulfhydryl groups and a scavenger of free radicals. The most established clinical application of NAC is to prevent fulminant hepatic failure caused by acetaminophen poisoning in treatments [20]. The application of NAC to research efforts for many diseases, such as cancer, human immunodeficiency virus (HIV) infection, and cardiovascular disease, has been reported [21, 22]. Besides, NAC has been shown to interact with many metabolic pathways, such as regulators of the cell cycle, apoptosis, gene expression, and signal transduction, as well as the cytoskeleton and immune system [23]. The mechanism of NAC in hepatitis amelioration includes not only the conventional pathway of reactive oxygen species (ROS) removal, but also other pathways such as NF-*κ*B and PI3K/Akt pathways [24]. We have previously demonstrated that NAC significantly alleviated ischemia-reperfusion induced acute liver injury, and a reduction of autophagy was observed in NAC pretreatment group in that model [25]. In the present study, we focus on the protective effects and probable mechanisms of NAC in concanavalin A-induced hepatitis in mice.

## 2. Materials and Methods

### 2.1. Reagents

N-Acetylcysteine (NAC) and ConA were purchased from Sigma–Aldrich (St. Louis, MO, USA). Enzyme-linked immunosorbent assay (ELISA) kits for detecting IL-2, IL-6, and TNF-*α* and IFN-*γ* were purchased from R&D Systems (Minneapolis, MN, USA). The antibodies used in this study include Akt, p-Akt, IL-2, IL-6, TNF-*α*, IFN-*γ*, NF-*κ*B, LC3II, Beclin 1, I*κ*B-*α*, and I*κ*B-*β* (Cell Signaling Technology, Beverly, MA, USA). The RNA PCR kit was purchased from Takara Biotechnology (Dalian, China).

### 2.2. Animals

Male BALB/c mice (6–8 weeks old, 22 ± 2 g) and male C57 mice (6–8 weeks old, 21 ± 2 g) were obtained from Shanghai Laboratory Animal Co. (SLAC, Shanghai, China). Mice were fasted in an environment at a temperature of 25 ± 2°C and 55% humidity with an alternating 12 h light–dark cycle. Mice were permitted free access to standard laboratory food and water. All experiments were performed according to the National Institutes of Health Guidelines and were also approved by the Animal Care and Use Committee of Shanghai Tongji University.

### 2.3. Experimental Design

ConA was dissolved in normal saline solution at a concentration of 20 mg/kg. Male BALB/c mice were randomly divided into one of four groups: group I (saline only) included 18 mice that were injected with saline via the tail vein, group II (ConA-treated) included 18 mice that were injected with ConA (20 mg/kg) via the tail vein, group III (2 h after NAC-treatment) included 18 mice that were injected with NAC (dissolved in saline, 150 mg/kg) which was administrated via the tail vein 2 h before injection with ConA via the tail vein, and group IV (before NAC-treatment) included 18 mice that were injected with NAC (dissolved in saline, 150 mg/kg) via the tail vein 30 min after they were injected with ConA via the tail vein. Male C57 mice were randomly divided into two groups: group A (ConA-treated) included 18 mice that were injected with ConA (20 mg/kg) via the tail vein and group B (NAC-treatment) included 18 mice administrated with NAC (dissolved in saline, 150 mg/kg) via the tail vein 2 h before ConA injection. We selected and sacrificed 6 mice from each group at 6, 12, and 24 h time points after ConA administration. At the end of the experiment, serum and liver tissue samples were obtained from each mouse and stored for further analysis.

### 2.4. Biochemical Analyses

#### 2.4.1. Analysis of Liver Enzymes

Serum was separated by centrifugation at 2000 rpm at room temperature for 10 min. To assess the level of hepatocellular injury after ConA-treatment, serum ALT and AST levels were measured using an automated chemistry analyzer (Olympus AU1000, Tokyo, Japan).

#### 2.4.2. Analysis of Serum Cytokines

To measure the serum levels of IL-2, IL-6, TNF-*α*, and IFN-*γ*, ELISA kits were used according to the manufacturer's instructions.

### 2.5. Histopathology

Mouse liver tissues were collected and stored in 4% paraformaldehyde for at least 24 h, and paraffin blocks were then prepared. Sections (3 *μ*m thick) were cut and stored at room temperature. Paraffin sections were stained with hematoxylin and eosin (H&E) to observe the level of inflammation and tissue damage by light microscopy.

### 2.6. Immunohistochemical Staining

Antigen was recovered in citrate buffer by incubation in a 95°C water bath for 20 min and then endogenous peroxidase activity was blocked by incubating in 3% hydrogen peroxide for 20 min at 37°C. Membranes were ruptured with 0.2% Triton at room temperature for 30 min and nonspecific binding sites were blocked with 5% BSA at 37°C for 20 min followed by room temperature incubation for 10 min. Liver slices were incubated overnight with rabbit anti-mouse p-AKT (1 : 500), rabbit anti-mouse LC3II (1 : 500), rabbit anti-mouse Beclin 1 (1 : 500), or rabbit anti-mouse NF-*κ*B (1 : 50). The next day, slices were incubated with secondary antibody (goat anti-rabbit; Epitomics, Burlingame, CA, USA) for 30 min at room temperature. Antibody binding analyses were performed using a DAB kit. Next, slides were counterstained with hematoxylin, dehydrated using a graded ethanol and xylene series, and mounted with Entellan. Slides were then observed by light microscopy. The assay was carried out using Image-Pro Plus software 6.0 (Media Cybernetics, Silver Spring, MD, USA). The frequency of NF-*κ*B-positive nuclei was also calculated using Image-Pro Plus 6.0. Three different fields of vision were randomly selected on one slide. We calculated the average of these three integrated optical densities (IOD). The same method was used with an additional two series for mice that were randomly selected from the same group. This method was applied on total groups.

### 2.7. Primary Cell Isolation

#### 2.7.1. Hepatocytes Isolation

Mouse hepatocytes were isolated by using a modified in situ collagenase perfusion technique. The portal vein of male Balb/c mice was cannulated after anesthesia and laparotomy by 20 mL prewarmed 37°C D-Hanks buffer, followed by 20 mL of 0.02% collagenase at a flow rate of 2 mL/min. After perfusion, liver tissues were removed and washed with 20 mL D-Hanks buffer. The capsule of the liver was removed, and hepatic tissues were dispersed and incubated in 20 mL of 0.01% collagenase in a shaking water bath at 37°C for approximately 30 min. After filtering through 60-mesh sterile nylon gauze, centrifuging at 500 rpm, and resuspending three times with RPMI-1640 culture medium, the hepatocytes were finally extracted and cultured in RPMI-1640 culture medium in a humidified incubator at 37°C and 5% CO_2_.

#### 2.7.2. Kupffer Cells Isolation

Liver tissues were washed with saline through portal vein 12 hours after Con A or PBS injection, then dissociated by 0.05% type IV collagenase for 30 min, centrifuged and then resuspended in RPMI-1640 culture medium, pipetted 10 mL into 50 mL tubes above 25 mL 25% Percoll, over 15 mL 50% Percoll, and then centrifuged at 2100 rpm/min for 15 min at 4°C. Kupffer cells at and above the third interface (between 25% and 50% Percoll solutions) were collected and centrifuged at 2100 rpm/min for 15 min at 4°C, washed twice by PBS, then resuspended in RPMI-1640 culture medium containing 10% FBS, and cultured in a 37°C incubator with 5% CO_2_ for 12 hours, removed spent medium, and was washed once by PBS and added in fresh medium; the remained adherent cells were Kupffer cells.

### 2.8. Immunofluorescence

The Isolated macrophages were first washed three times with PBS solution for 5 min and then fixed with 4% paraformaldehyde for 10 min. The cells were then permeabilized in 0.3% Triton X-100 (Sigma, USA) for 10 min and washed with PBS solution. The macrophages were blocked with 5% bovine serum albumin (BSA) in PBS for 1 h at room temperature and incubated with rabbit anti-mouse IL-2 antibody (diluted 1 : 100), rabbit anti-mouse IL-6 antibody (diluted 1 : 100), rabbit anti-mouse TNF-*α* antibody (diluted 1 : 100), and rabbit anti-mouse IFN-*γ* antibody (diluted 1 : 100) overnight at 4°C. Next day, the slides were washed with PBS solution again and incubated with appropriate fluorescein isothiocyanate-conjugated secondary antibody for 1 h. The secondary antibody was subsequently washed and the slides were incubated with 2-(4-amidinophenyl)-6-indolecarbamidine dihydrochloride (DAPI) (Life Technologies, USA) 3 min for nuclear staining and finally visualized with LSM710 Carl Zeiss confocal microscope (Carl Zeiss AG, Germany).

### 2.9. Western Blot Analysis

Liver tissues were recovered from −80°C storage and rapidly ground in liquid nitrogen before lysis in RIPA lysis buffer with protease inhibitors. Protein concentrations were detected using the BCA method. Equivalent amounts of total protein (120 *μ*g) were boiled and subjected to sodium dodecyl sulfate–polyacrylamide gel electrophoresis (SDS–PAGE) and then transferred onto a Polyvinylidene fluoride (PVDF) membrane. Nonspecific binding was blocked with 5% nonfat milk (dissolved in PBS) for 1 h and then blots were incubated overnight at 4°C with rabbit anti-mouse IL-2 (1 : 500), rabbit anti-mouse IL-6 (1 : 500), rabbit anti-mouse TNF-*α* (1 : 500), mouse anti-mouse *β*-actin (1 : 1000), rabbit anti-mouse LC3II (1 : 500), rabbit anti-mouse Beclin 1 (1 : 500), or rabbit anti-mouse IFN-*γ* (1 : 500) antibodies diluted in 5% milk. *β*-actin was used as an internal reference for cytoplasmic proteins. All PVDF membranes were washed with PBST (PBS containing 0.1% Tween 20), then were incubated with a secondary goat anti-mouse or anti-rabbit antibody (1 : 1000), and dissolved in PBST for 45 min at 37°C. Finally, membranes were washed with PBST three times for 5 min and proteins were detected using an Odyssey two-color infrared laser imaging system (detected based on fluorescence).

### 2.10. Reverse Transcription-Polymerase Chain Reaction (RT-PCR) and Quantitative RT-PCR (qRT-PCR)

In this study, mRNA transcription of liver tissue was detected and analyzed via quantitative RT-PCR (qRT-PCR). Total RNA was extracted from frozen liver tissue using TRIzol reagent (Takara, Shiga, Japan) following the manufacturer's instructions. To measure the expression of target genes in the liver, qRT-PCR was performed using a 7900HT Fast Real-Time PCR system (ABI, Foster City, CA, USA) according to the manufacturer's instructions and using SYBR Premix EX Taq (TaKaRa Biotechnology, China). The primer sequences used for this experiment are shown in Table 1.

### 2.11. Transmission Electron Microscopy (TEM)

Following the harvesting of liver tissues, a portion of mouse liver tissue was placed in 4% glutaraldehyde and then fixed in 1% OsO_4_. Cells were observed by TEM (JEOL, JEM 1230).

### 2.12. Statistical Analyses

Data are expressed as means ± SD. Date of ELISA, real-time PCR, levels of ALT, AST, necrotic area, and western blot and immunohistochemistry were analyzed using one-way analysis of variance (ANOVA) and Student's *t*-test. ANOVA was used in groups and Student's *t*-test on the statistical analyses applied in two groups separately. In all comparisons, *P* < 0.05 was considered to indicate a statistically significant difference. All statistical analyses were performed using SPSS 17.0 for Windows (Chicago, IL, USA).

## 3. Results

### 3.1. NAC Protects Mice from ConA-Induced Liver Injury When Administered before or after ConA Injection

**Figure 1:**
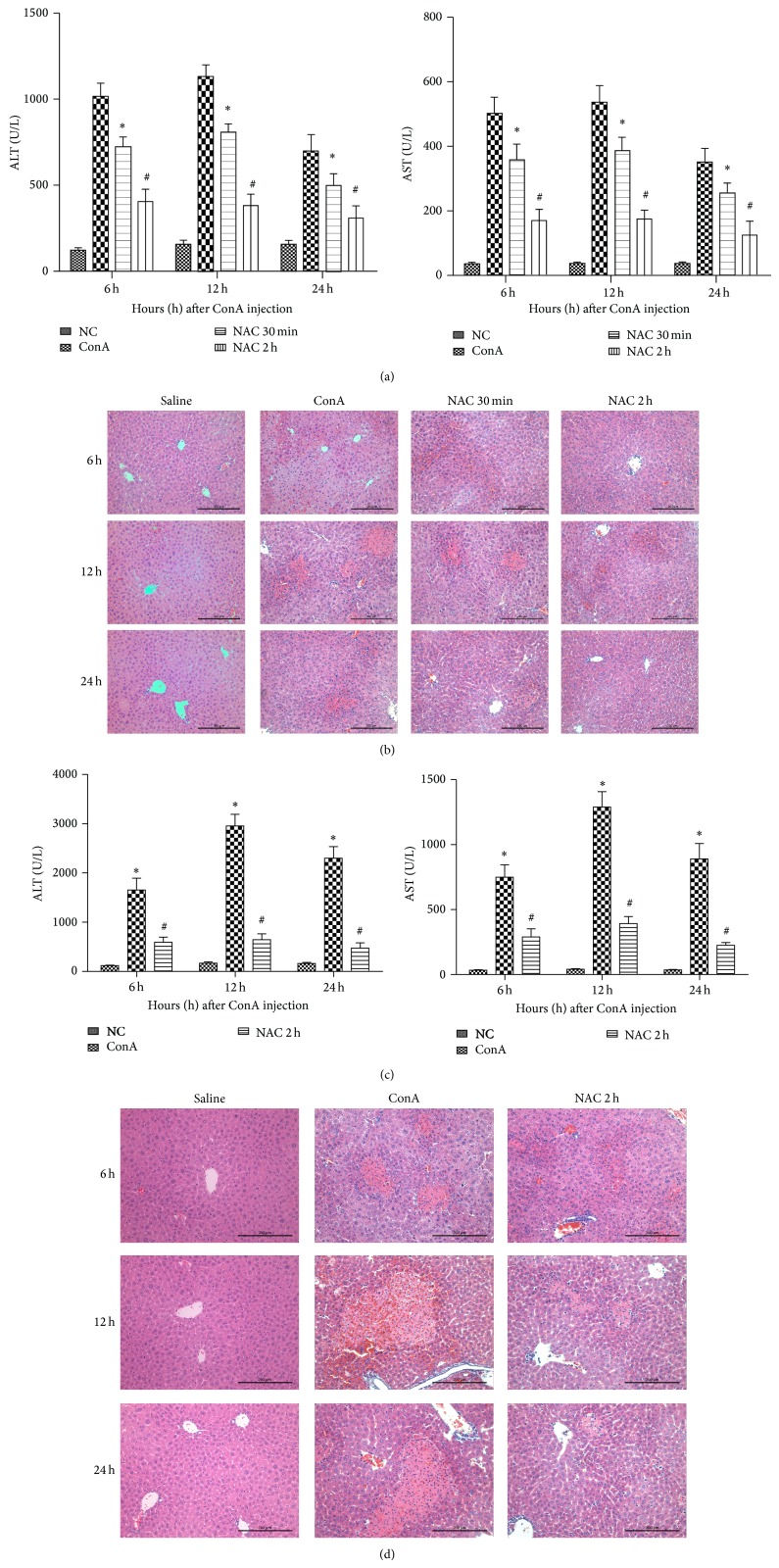
Effect of NAC on ConA-induced autoimmune hepatitis. (a) Effects of NAC on plasma ALT and AST levels at 6 h, 12 h, and 24 h after ConA injection in BALB/c mice. Data are expressed as mean ± SD (*n* = 6; ^*^ *P* < 0.05 for ConA versus NAC 30 min (post-NAC-treated, 30 min after injection with ConA); ^#^ *P* < 0.05 for NAC 30 min (post-NAC-treated, 30 min after injection with ConA) versus NAC 2 h (NAC-pretreated, 2 h before injection with ConA)). (b) Photomicrographs of representative livers collected 6, 12, and 24 h after ConA injection and stained with hematoxylin and eosin (H&E), ×200 magnification. (c) Effects of NAC on plasma ALT and AST levels at 6 h, 12 h, and 24 h after ConA injection in C57 mice. Data are expressed as mean ± SD (*n* = 6; ^*^ *P* < 0.05 for ConA versus control group; ^#^ *P* < 0.05 for ConA versus NAC 2 h (NAC-pretreated, 2 h before injection with ConA)). (d) In C57 mice, photomicrographs of representative livers collected 6, 12, and 24 h after ConA injection and stained with hematoxylin and eosin (H&E), ×200 magnification.

The serum ALT and AST levels increased (AST, 410 ± 112 U/L; ALT, 850 ± 226 U/L) in ConA model group when compared to the both NAC-treated groups (AST, 130 ± 12 U/L; ALT, 300 ± 24 U/L in NAC-pretreatment; AST, 130 ± 12 U/L; ALT, 300 ± 24 U/L in post-NAC-treatment) (Figure 1(a)) at 3 time points as designed. The control groups showed much lower levels of aminotransferase (AST, 53 ± 17 U/L; ALT, 120 ± 28 U/L). The histopathological changes were consistent with the AST and ALT results. We observed massive areas of necrosis in the ConA-alone group (Figure 1(b)). By contrast, the NAC-treated groups showed minor liver damage, indicating that NAC-treatment significantly reduced liver necrosis. The NAC pretreatment group showed the most effective protection. Based on a statistical analysis using Image-Pro Plus 6.0 software, we detected a robust statistically significant difference between the different groups. Considering the BALB/c mice are relatively resistant to ConA hepatitis, we used C57 mice, a strain relatively susceptible to ConA hepatitis strain, to test the effectiveness of NAC. Indeed, the ConA-treated groups showed a more serious injury compared with BALB/c mice, and NAC still exhibited effective therapy effect (Figures 1(c) and 1(d)). These results indicated that NAC-treatment ameliorated ConA-induced immune hepatitis in mice.

### 3.2. NAC Inhibits Cytokine Release in ConA-Induced Hepatitis

**Figure 2:**
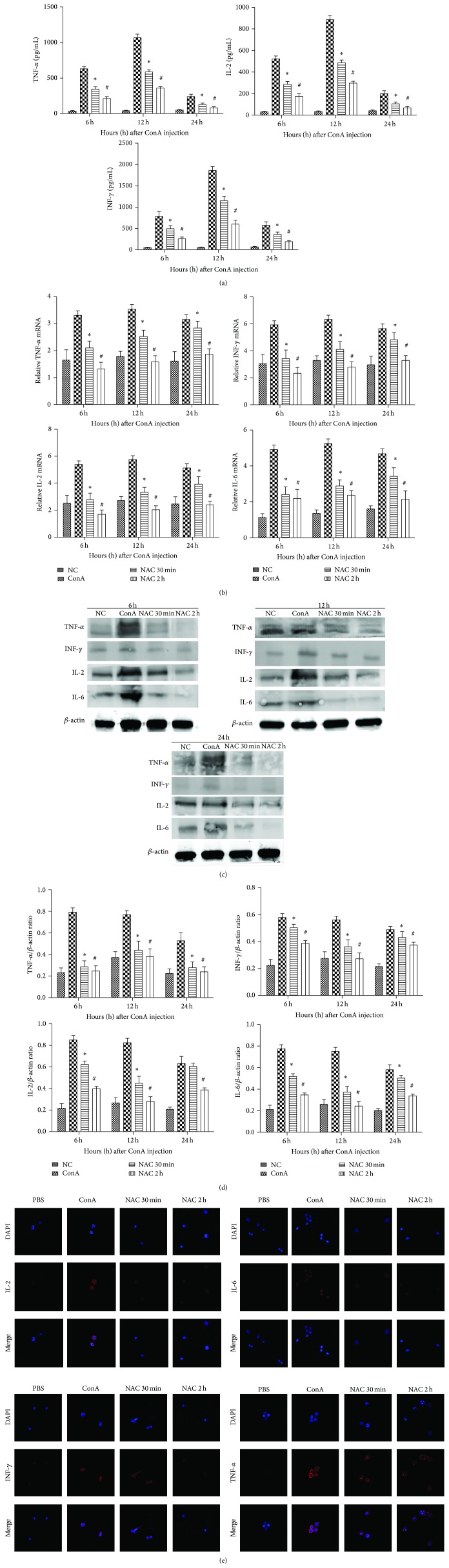
NAC inhibits cytokines release in ConA-induced autoimmune hepatitis. (a) Serum levels of IL-2, IFN-*γ*, and TNF-*α* were measured by ELISA at 6 h, 12 h, and 24 h after ConA injection in mice. Data are expressed as mean ± SD (*n* = 6; ^*^ *P* < 0.05 for ConA versus NAC 30 min (post-NAC-treated, 30 min after injection with ConA); ^#^ *P* < 0.05 for NAC 30 min (post-NAC-treated, 30 min after injection with ConA) versus NAC 2 h (NAC-pretreated, 2 h before injection with ConA)). (b) The expressions of IL-2, IL-6, IFN-*γ*, and TNF-*α* on mRNA levels were detected by real-time PCR. Data are expressed as mean ± SD (*n* = 6; ^*^ *P* < 0.05 for ConA versus NAC 30 min; ^#^ *P* < 0.05 for NAC 30 min versus NAC 2 h). (c) The expressions of IL-2, IL-6, IL-1*β*, IFN-*γ*, and TNF-*α* on protein levels were detected with western blot. (d) The results of western blot were analyzed with Quantity One. Data are expressed as mean ± SD (*n* = 6; ^*^ *P* < 0.05 for ConA versus NAC 30 min; ^#^ *P* < 0.05 for NAC 30 min versus NAC 2 h). (e) The differently expressions of IL-2, IL-6, IFN-*γ*, and TNF-*α* in macrophages' localization were evaluated by immunofluorescence (magnification is 630x).

A number of proinflammatory cytokines play key roles in the progression of hepatitis, such as IL-2, IFN-*γ*, and TNF-*α*. Therefore, serum levels of IL-2, IFN-*γ*, and TNF-*α* were measured by ELISA in the control, ConA-treated, NAC-pretreated, and post-NAC-treated groups (Figure 2(a)). Our result shows that the levels of these cytokines increased in the ConA-treated group after ConA induction and peaked at 12 h. Additionally, as expected, the production of IL-2, IFN-*γ*, and TNF-*α* was reduced after NAC-treatment, as seen at 6 and 12 h, especially in the NAC-pretreated group. Furthermore, to confirm our observations, mRNA and protein expression levels of IL-2, IL-6, IFN-*γ*, and TNF-*α* were detected by real-time PCR and western blotting, respectively, at the designed time points (Figures 2(b) and 2(c)). We found that IL-2, IL-6, IFN-*γ*, and TNF-*α* were significantly increased in the ConA-treated group and were diminished in the NAC-treated groups for both mRNA and protein expression levels at all three time points. Additionally, IL-2, IL-6, IFN-*γ*, and TNF-*α* expression peaked at 12 h, indicating that these cytokines were most robustly expressed during the early phase of ConA-induced hepatitis. Additionally, the western blot results were analyzed using Quantity One, indicating that these changes in levels were statistically significant (Figure 2(d)). In addition, we performed immunofluorescence test of macrophages to observe the changes of inflammatory cytokines. The immunohistochemistry graphs show that IL-2, IL-6, IFN-*γ*, and TNF-*α* were increased in the ConA-treated group and were diminished in the NAC-treated groups. Thus, treatment with NAC attenuated the release of ConA-induced hepatitis-associated cytokines, such as IL-2, IL-6, IFN-*γ*, and TNF-*α*.

### 3.3. The Effect of NAC on Akt Expression in ConA-Induced Hepatitis in Mice

**Figure 3:**
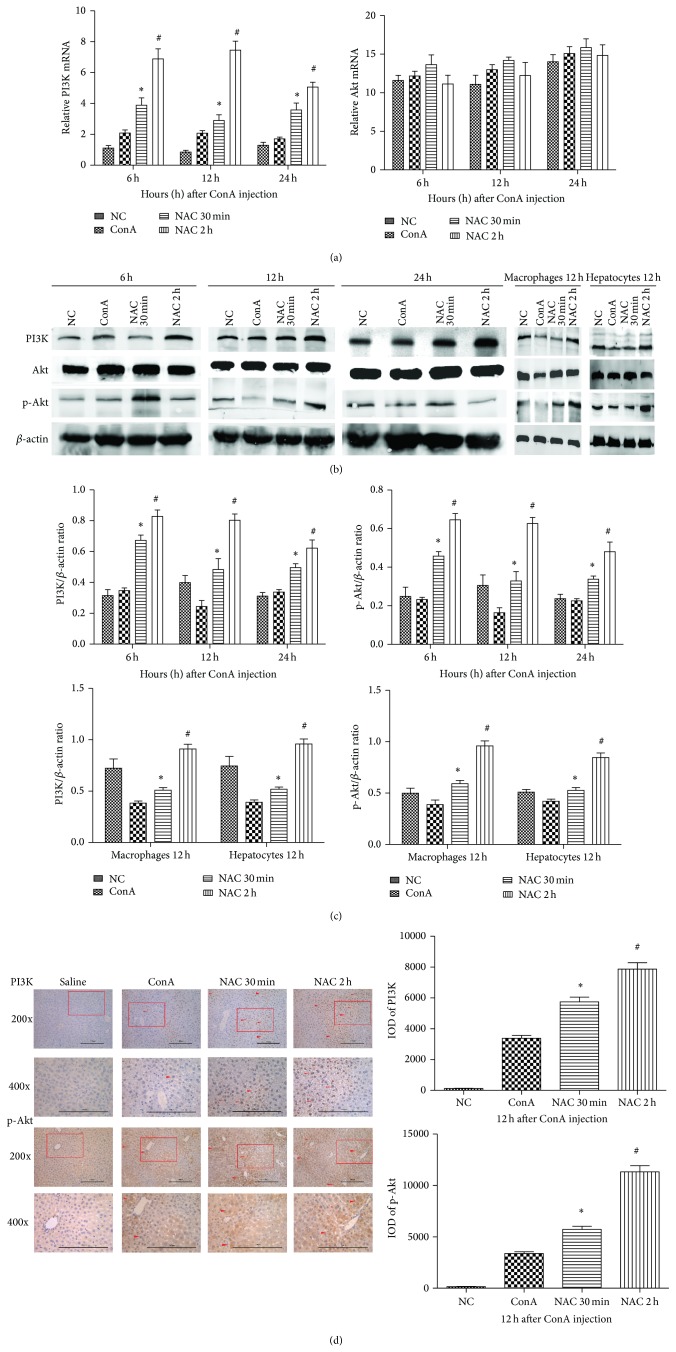
The effect of NAC on expression of PI3K/Akt pathway in ConA-induced autoimmune hepatitis. (a) The expression of PI3K and Akt on mRNA levels was detected by real-time PCR. Data are expressed as mean ± SD (*n* = 6; ^*^ *P* < 0.05 for ConA versus NAC 30 min; ^#^ *P* < 0.05 for NAC 30 min versus NAC 2 h). (b) The expressions of PI3K, p-Akt, and Akt on protein levels were detected with western blot. The expressions of PI3K, p-Akt, and Akt on protein levels of macrophages and hepatocytes were detected by western blot at 12 h time point. (c) The results of western blot were analyzed with Quantity One. Data are expressed as mean ± SD (*n* = 6; ^*^ *P* < 0.05 for ConA versus NAC 30 min; ^#^ *P* < 0.05 for NAC 30 min versus NAC 2 h). (d) The expressions of PI3K and p-Akt on protein levels were detected by immunohistochemistry staining in hepatic tissues at 12 h (×200 and ×400 magnification). The positive cells were indicated with red arrows. The result was analyzed using Image-Pro Plus 6.0. Date are showed as mean ± SD (*n* = 6; ^*^ *P* < 0.05 for ConA versus NAC 30 min; ^#^ *P* < 0.05 for NAC 30 min versus NAC 2 h).

We further characterized the possible mechanism whereby NAC ameliorates ConA-induced hepatitis. It has been reported that activation of the Akt pathway can reduce the severity of ConA-induced hepatitis. Therefore, we asked whether NAC acts by activating the Akt pathway in ConA-induced hepatitis. The mRNA expression levels of Akt and PI3K were measured by qRT-PCR, and we found that the expression of total Akt showed no obvious change in all groups and time points, while the expression of PI3K increased in the NAC-treated groups compared to the ConA-treated group. This result was supported by measurements of protein expression of total Akt, p-Akt, and PI3K, as determined by western blotting. The total protein levels of Akt showed no obvious change in the ConA-treated, NAC-treated, or control groups, and also there was no significant change for all time points (Figure 3(b)). The expression of p-Akt and PI3K was significantly increased in the NAC-treated groups compared to the ConA-treated group. These data were analyzed using Quantity One, indicating that there was a statistically significant difference among these changes (Figure 3(c)). This finding indicated that NAC clearly upregulated the Akt pathway by phosphorylation the Akt protein. We extracted the macrophages and hepatocytes to observe the protein expression of PI3K/Akt pathway in 12 hours after ConA injection (Figures 3(c) and 3(d)). The results are consistent with the tissue experimental results. To further confirm these observations, we measured the expression levels of proteins in the Akt pathway in liver tissues using immunohistochemistry. As expected, our findings supported the conclusions mentioned above (Figure 3(d)). The statistical analysis was carried out using Image-Pro Plus 6.0 software, and we clearly detected a statistically significant difference among the different groups. Based on these results, we conclude that NAC-treatment can ameliorate ConA-induced hepatitis at least in part through regulation of the Akt pathway.

### 3.4. The Effects of NAC on the NF-*κ*B Pathway in ConA-Induced Hepatitis in Mice

**Figure 4:**
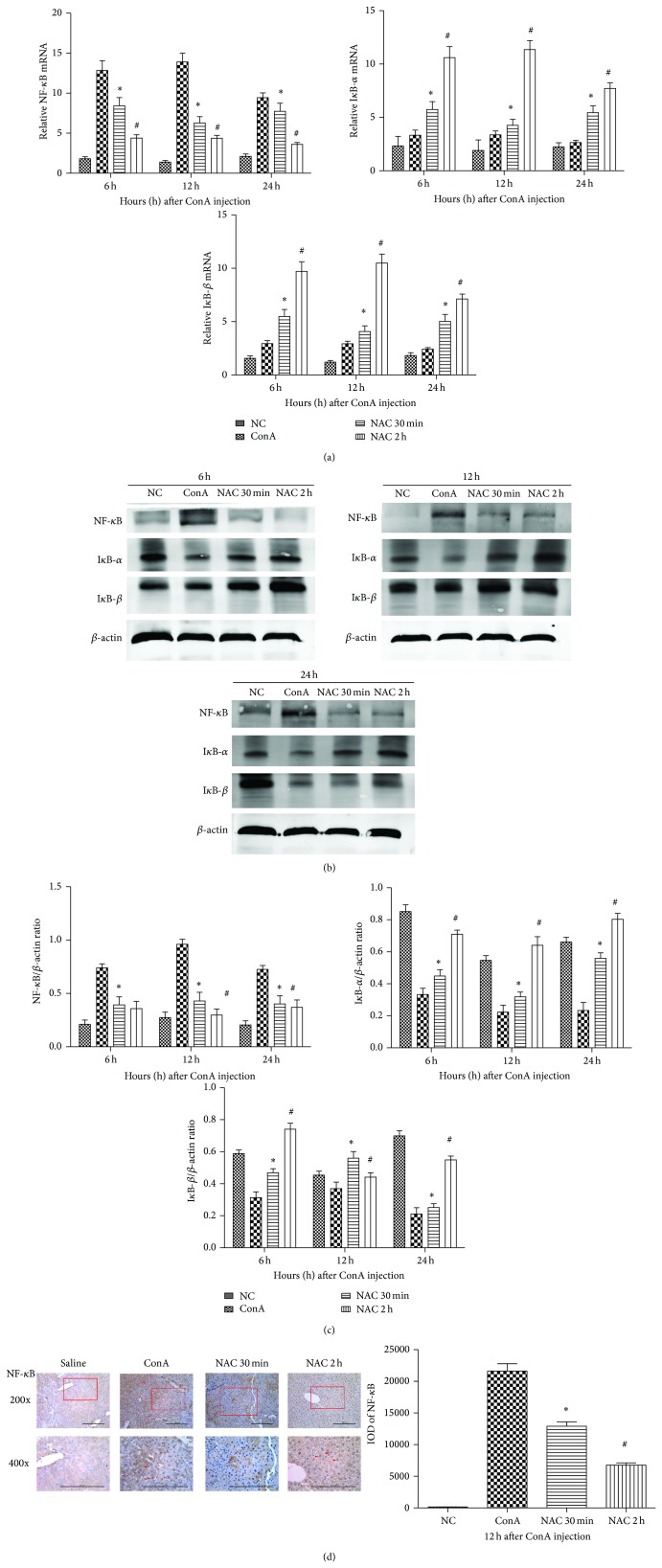
The effect of NAC on NF-*κ*B pathway in ConA-induced hepatitis in mice. (a) The expressions of NF-*κ*B, I*κ*B-*α*, and I*κ*B-*β* on mRNA levels were detected by real-time PCR. Data are expressed as mean ± SD (*n* = 6; ^*^ *P* < 0.05 for ConA versus NAC 30 min; ^#^ *P* < 0.05 for NAC 30 min versus NAC 2 h). (b) The expressions of NF-*κ*B, I*κ*B-*α* and I*κ*B-*β* on protein levels were detected with western blot. (c) The results of western blot were analyzed with Quantity One. Data are expressed as mean ± SD (*n* = 6; ^*^ *P* < 0.05 for ConA versus NAC 30 min; ^#^ *P* < 0.05 for NAC 30 min versus NAC 2 h). (d) The expressions of NF-*κ*B on protein levels were detected by immunohistochemistry staining in hepatic tissues at 12 h (×200 and ×400 magnification). The positive cells were indicated with red arrows. The result was analyzed using Image-Pro Plus 6.0. Date are showed as mean ± SD (*n* = 6; ^*^ *P* < 0.05 for ConA versus NAC 30 min; ^#^ *P* < 0.05 for NAC 30 min versus NAC 2 h).

NF-*κ*B plays a key role in the activation of several proinflammatory cytokines. To determine whether NAC inhibited the NF-*κ*B pathway in ConA-induced hepatitis, we measured the mRNA expression levels of NF-*κ*B, I*κ*B-*α*, and I*κ*B-*β* by real-time PCR (Figure 4(a)). We measured the protein expression levels of NF-*κ*B, I*κ*B-*α*, and I*κ*B-*β* by western blotting. We found that NAC-treatment reduced the degradation of I*κ*B-*α* and I*κ*B-*β* in protein level, especially at 12 h, and NAC-treatment also reduced the expression levels of NF-*κ*B (Figure 4(b)). Furthermore, we explored the expression of proteins in the NF-*κ*B pathway in the liver tissue using immunohistochemistry. We found that NF-*κ*B was mostly expressed and located in nuclei in the ConA-induced compared to the control group. Additionally, we found that the expression of NF-*κ*B in nuclei was obviously reduced in the NAC-treated groups, especially at 12 h (Figure 4(c)). We analyzed these data with Image-Pro Plus 6.0 software, and statistically significant differences between the treatment groups had been detected (Figure 4(d)).

### 3.5. The Effect of NAC on Autophagy in ConA-Induced Hepatitis

**Figure 5:**
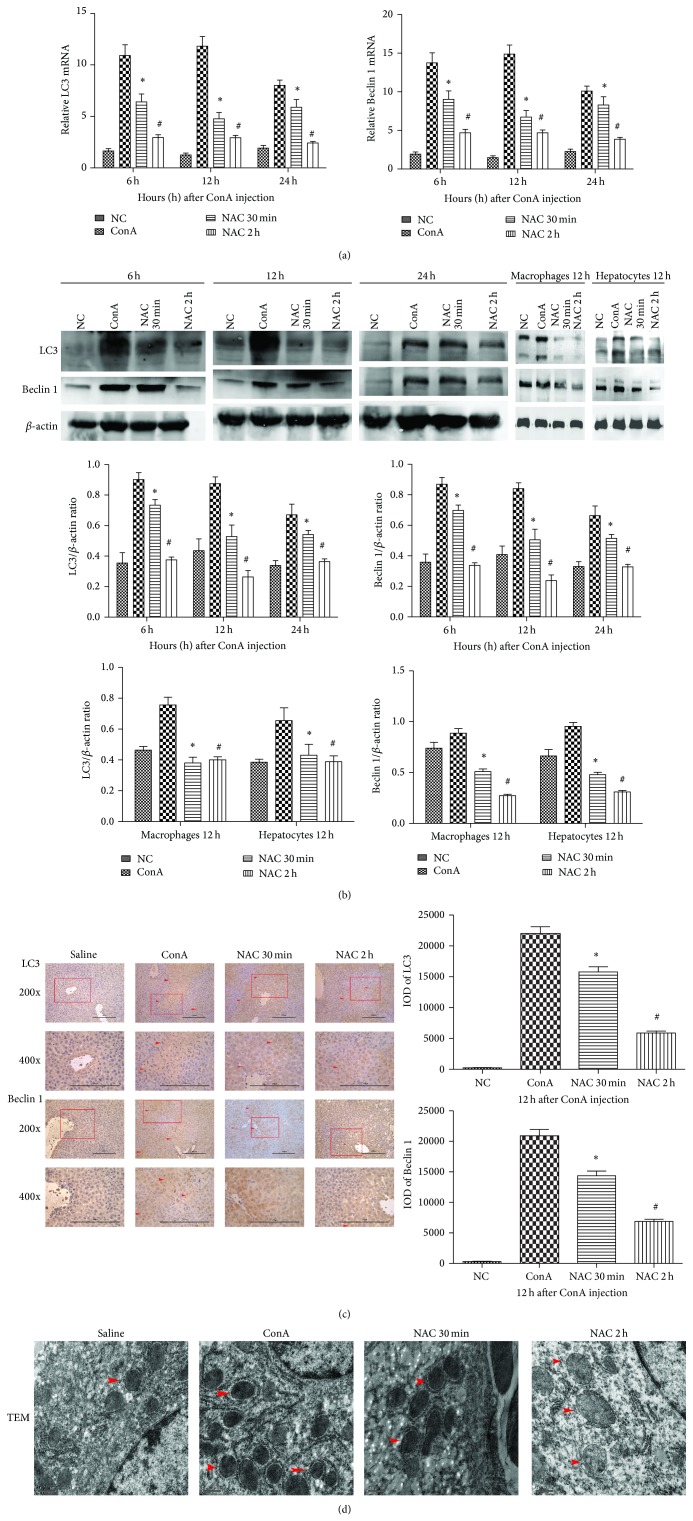
The effect of NAC on autophagy in ConA-induced hepatitis. (a) The expressions of LC3 and Beclin 1 on mRNA levels were detected by real-time PCR. Data are expressed as mean ± SD (*n* = 6; ^*^ *P* < 0.05 for ConA versus NAC 30 min; ^#^ *P* < 0.05 for NAC 30 min versus NAC 2 h). (b) The expressions of LC3 and Beclin 1 on protein levels were detected with western blot. The expressions of LC3 and Beclin 1 on protein levels of macrophages and hepatocytes were detected by western blot at 12 h time point. The results of western blot were analyzed with Quantity One. Data are expressed as mean ± SD (*n* = 6; ^*^ *P* < 0.05 for ConA versus NAC 30 min; ^#^ *P* < 0.05 for NAC 30 min versus NAC 2 h). (c) The expressions of LC3 and Beclin 1 on protein levels were detected by immunohistochemistry staining in hepatic tissues at 12 h (×200 and ×400 magnification). The positive cells were indicated with red arrows. The result was analyzed using Image-Pro Plus 6.0. Date are showed as mean ± SD (*n* = 6; ^*^ *P* < 0.05 for ConA versus NAC 30 min; ^#^ *P* < 0.05 for NAC 30 min versus NAC 2 h). (d) Morphology of autophagosomes in hepatocytes at 12 h detected by electron microscopy (×20000 magnification).

ConA-induced hepatitis appeared to increase autophagosome content in cells. To evaluate the effects of NAC on autophagy in ConA-induced hepatitis, we measured the levels of LC3II and Beclin 1 by real-time PCR and western blotting. LC3II is a marker of autophagy activation that plays a significant role in regulating autophagy. We detected changes in LC3II and Beclin 1 cDNA levels by real-time PCR (Figure 5(a)). We detected a statistically significant increase in the ConA-treated group compared to the control and NAC-treated groups (*P* < 0.05). The protein expression levels, which were detected by western blotting, were in accordance with the above results (Figure 5(b)). The protein expressions of both two autophagy makers were also learned in macrophages and hepatocytes in 12 hours after ConA injection (Figure 5(b)). The results showed a consistency with the tissue experimental results. To further confirm this observation, we assessed the protein expression levels of LC3 and Beclin 1 in liver tissues using immunohistochemistry (Figure 5(c)). We observed high expression levels of LC3 and Beclin 1 in the ConA-treated group compared to the control group. NAC downregulate the expression of LC3 and Beclin 1, which could be observed in the NAC-treated groups. These data were analyzed using Image-Pro Plus 6.0 software, and we detected a statistically significant difference among the groups. It is also important to note the morphology of the autophagosome during autophagy, which we assessed using TEM to determine the ultrastructure of hepatocyte. The ConA-treated group showed uneven nuclear chromatin distribution, with partial agglutination. Additionally, lysosomes and autophagosome were obviously increased (Figure 5(d)). In the NAC-treated group, destruction of the cells was obviously reduced, as was the number of agglutinations, and the mitochondrial structural integrity was improved.

## 4. Discussion

Hepatitis remains a global health problem, which consist of viral hepatitis, toxic liver disease, alcoholic liver disease, and autoimmune hepatitis, of which only a portion can be treated pharmacologically [26]. Additionally, a large amount of evidence supports a relationship between hepatitis and hepatocellular carcinoma. Therefore, novel pharmaceuticals need to be developed and tested to provide more effective therapies in the clinic. NAC is a thiol and a precursor of L-cysteine and GSH that has potential health benefits. In this study, we demonstrated that ConA-induced hepatitis was ameliorated in NAC-treated mice.

Hepatitis, especially immune hepatitis, is mainly caused by the activation of immune cells. Mice injected with the T-cell mitogen plant lectin ConA exhibit T lymphocyte activation [10, 13]. Previous studies have demonstrated that the pathogenesis of ConA-induced hepatitis mainly occurs as a consequence of inflammatory cytokine release [27]. Proinflammatory cytokines play important roles in hepatitis, including TNF-*α*, IFN-*γ*, IL-2, and IL-6. In this study, PCR and ELISA analysis revealed that the mRNA expression levels of TNF-*α*, IFN-*γ*, IL-2, and IL-6 in the liver of ConA-treated mice were significantly upregulated at three time points. In the NAC-treated groups, we could observe the downregulation of inflammatory cytokine expression. Therefore, we confirmed that NAC could inhibit the expression of those inflammatory cytokines. One of the major consequences of reduction of these proinflammatory cytokines was the difference of serum ALT and AST levels, which were considered to be one metric to assess the severity of liver injury, in ConA-treated group and NAC-treated groups. Besides, the histology change of liver, observed by H&E staining, corroborated the serological changes, shown in Figure 1.

NF-*κ*B is a key regulator of the expression of a variety of genes involved in immune and inflammatory responses [28]. It can potently induce proinflammatory mediators and plays an important role in the development of ConA-induced hepatitis [29, 30]. In this study, we found that NAC can inhibit the degradation of I*κ*B-*α* and impede the nuclear translocation of NF-*κ*B. Increased protein of expression of I*κ*B-*α* and I*κ*B-*β* and reduced expression of NF-*κ*B were observed in NAC pretreatment groups, shown in Figure 4. The combination of I*κ*B-*α*, *β* with NF-*κ*B will inhibit the translocation of NF-*κ*B into nucleus. As a result, proinflammation cytokines expression was reduced [31]. Therefore, we speculated that NAC attenuating ConA induced inflammation by reduction of I*κ*B-*α* and I*κ*B-*β* degradation. Similar results have been published by other researchers [32, 33].

We also explored autophagic cell death, one important form of ConA-induced hepatitis [34]. We observed that ConA-induced hepatitis was associated with obvious autophagic cell death, and in the NAC-treated groups we observed that autophagic cell death was significantly inhibited. Autophagic cell death is characterized by excessive lysosome-mediated degradation of cellular contents. Autophagy is regulated by many regulators; one of the most important ones is the mTOR pathway, which can be controlled by the PI3K/Akt pathway [14]. We measured the expression of Beclin 1 and LC3 to explore the effects of NAC in ConA-induced autophagy. The levels of Beclin 1 and LC3II in the NAC-treated group were significantly reduced. Compared to the NAC-treated and control groups, the levels of Beclin 1 and LC3II in the ConA-treated group were significantly increased. This finding indicates that NAC can mitigate ConA-induced autophagy and liver injury and suggest that the inhibition of autophagy may represent a new therapy for autoimmune hepatitis. It has been well established that the PI3K/Akt pathway can regulate cell proliferation, apoptosis, differentiation, and senescence. It has been also reported that ConA treatment can reduce Akt phosphorylation and PI3K activity, indicating the downregulation of the PI3K/Akt pathway. In the NAC-treated group, there was significantly increased expression of p-Akt. This investigation may further support that the supplementation of NAC enhances hepatocyte PI3K/Akt pathway activation and leads to further suppression of autophagic cell death.

## 5. Conclusions

Our results described a new pharmaceutical to provide more effective therapies for immune hepatitis. Our results are as follows: (1) NAC can attenuate ConA-induced immune hepatitis in BALB/c mice; (2) NAC reduces TNF-*α*, IL-2, IL-6, and IFN-*γ* expression and inhibits NF-*κ*B activation in ConA-induced hepatitis; (3) NAC inhibits autophagy in ConA-induced immune hepatitis by activating PI3K/AKT signaling pathway.

## Figures and Tables

**Table 1:** 

Gene	Primer sequence (5′→3′)
IL-2	
Forward	TGAGCAGGATGGAGAATTACAGG
Reverse	GTCCAAGTTCATCTTCTAGGCAC
*β*-actin	
Forward	GGCTGTATTCCCCTCCATCG
Reverse	CCAGTTGGTAACAATGCCATGT
Beclin 1	
Forward	ATGGAGGGGTCTAAGGCGTC
Reverse	TGGGCTGTGGTAAGTAATGGA
LC3	
Forward	GACCGCTGTAAGGAGGTGC
Reverse	AGAAGCCGAAGGTTTCTTGGG
NF-*κ*B	
Forward	ATGGCAGACGATGATCCCTAC
Reverse	CGGATCGAAATCCCCTCTGTT
IL-6	
Forward	CTGCAAGAGACTTCCATCCAG
Reverse	AGTGGTATAGACAGGTCTGTTGG
TNF-*α*	
Forward	CAGGCGGTGCCTATGTCTC
Reverse	CGATCACCCCGAAGTTCAGTAG
INF-*γ*	
Forward	ATGAACGCTACACACTGCATC
Reverse	CCATCCTTTTGCCAGTTCCTC
PI3K	
Forward	ACACCACGGTTTGGACTATGG
Reverse	GGCTACAGTAGTGGGCTTGG
Akt	
Forward	ATGAACGACGTAGCCATTGTG
Reverse	TTGTAGCCAATAAAGGTGCCAT
I*κ*B-*α*	
Forward	GTCAGGACCGTGTTCTCAAGG
Reverse	GCTTCTTTGATGTTACTGAGGGC
I*κ*B-*β*	
Forward	CTGAAGATCGCCTGTAGCAAA
Reverse	TCCATCTGTAACCAGCTCCAG
